# TIME HORIZONS VERSUS TIME SAVORING: WHICH BEST PREDICTS AGE-RELATED IMPROVEMENTS IN EMOTIONAL WELL-BEING?

**DOI:** 10.1093/geroni/igac059.754

**Published:** 2022-12-20

**Authors:** Tyler Matteson, Li Chu, Laura Carstensen

**Affiliations:** Stanford University, Stanford, California, United States; Stanford University, Menlo Park, California, United States; Stanford University, Stanford, California, United States

## Abstract

Previous research has shown that time horizons, as measured by the future time perspective (FTP) scale, yields mixed findings about the relationship between perceived time and emotional well-being. Expansive time horizons often predict better well-being than limited time horizons, raising questions about a core postulate of socioemotional selectivity theory (SST). The present study introduces a more nuanced construct about perceived time and introduces a new scale, time savoring, which captures the heightened value of time as time becomes more limited. Based on 1,384 participants (Mage = 54.55, age range = 18-96), time savoring and age are positively correlated (r(1,382) = .23, p < .001). Time savoring partially mediates age and well-being (i.e., greater happiness, lower depressive symptoms, and higher life satisfaction). Parallel mediation with multiple mediators demonstrated the measure’s discriminant validity from FTP and personality measures (e.g., agreeableness and conscientiousness), offering a promising way to measure heightened value of time.

